# Bipolar Dislocation of the Clavicle: A Report of Two Cases with Different Injury Patterns and a Literature Review

**DOI:** 10.1155/2017/2935308

**Published:** 2017-12-28

**Authors:** Ichiro Okano, Takatoshi Sawada, Katsunori Inagaki

**Affiliations:** ^1^Department of Orthopaedic Surgery, Ohta-Nishinouchi Hospital, 2-5-20 Nishinouchi, Koriyama, Fukushima 963-8558, Japan; ^2^Department of Orthopaedic Surgery, Showa University School of Medicine, 1-5-8 Hatanodai, Shinagawa-ku, Tokyo 142-8666, Japan

## Abstract

Bipolar dislocation of the clavicle is a rare injury that is defined as a concomitant dislocation of the ipsilateral acromioclavicular joint and sternoclavicular joint. This injury is also described as a floating clavicle. Although this injury has been known for nearly two centuries, knowledge about it is limited and the treatment strategy remains controversial. Bipolar dislocation includes several combinations of both joints' injury types. We reported two patients with bipolar dislocation of the clavicle: one with an anterior dislocation and the other with a posterior dislocation of the sternoclavicular joint. After reviewing the currently available literature, we discussed these cases to highlight the necessity of a specific treatment approach that is modified based on the pattern of each joint's lesion.

## 1. Introduction

Bipolar dislocation of the clavicle is a rare injury that was first described in 1831 by Porral, and it is characterized as dislocation of both ends of the clavicle: the acromioclavicular joint (ACJ) and sternoclavicular joint (SCJ). This injury is also called “floating clavicle” [1–7], although this name is often used to describe any combinations of dislocation and fracture at both ends of the clavicle [2, 5]. In 1924, Beckman published a case report with a review of 15 previously reported patients with this injury, but no other cases were reported until the early 1980s. Even after 1980, fewer than 30 clinical cases were found published in the English literature. Information about the diagnosis, treatment, and prognosis of patients with bipolar dislocation of the clavicle remains limited. Some authors recommended operative treatment for young, high-demand patients [7–13], but others chose conservative treatment for these patients and reported good results [6, 14–16]. In this report, we described two patients with surgically treated bipolar dislocation of the clavicle; each patient had different injury patterns of the SCJ. We also reviewed the recently published literature and discussed the characteristics of this rare injury and treatment strategy for it.

## 2. Case Presentation

### 2.1. Case 1

**Figure 1:**
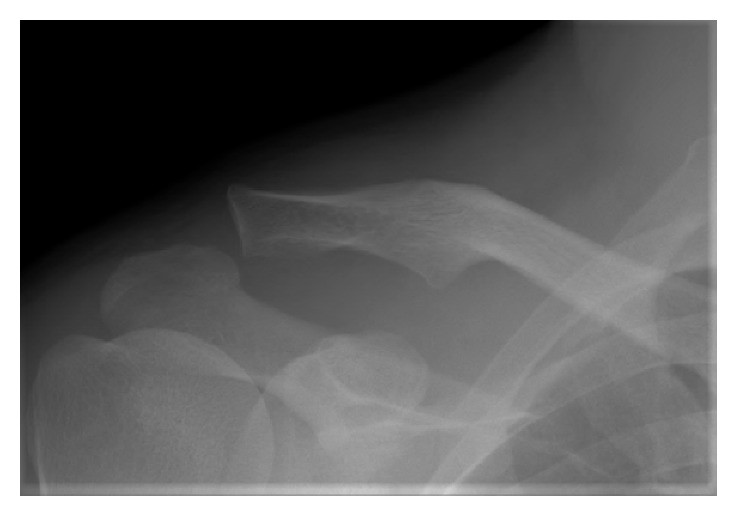
Case 1: images at the first presentation. A radiograph shows type III ACJ dislocation.

**Figure 2:**
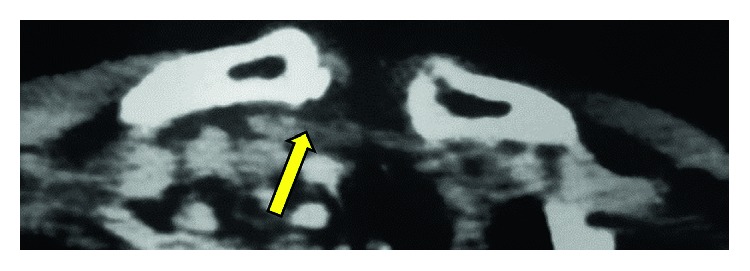
Case 1: a computed tomography scan shows anterior SCJ dislocation (arrow).

A 45-year-old East Asian, right hand-dominant male carpenter fell from a ladder and landed on his right shoulder. He was brought to a local clinic and complained of pain in his right shoulder. A physical examination demonstrated swelling around both ends of the right clavicle, but neurovascular symptoms were not observed. Initial radiographs and a computed tomography (CT) scan of the right clavicle showed type III ACJ dislocation (Figure 1) and anterior SCJ dislocation (Figure 2). The patient was diagnosed with bipolar dislocation of the clavicle. Examination of the trauma also showed a slight right-sided hemopneumothorax and fracture of the right seventh rib, which were treated conservatively.

A modified Cadenat's procedure [17] was performed for the ACJ dislocation 10 days later. Kirschner wires were placed for 8 weeks, and after the implant was removed, full range of motion (ROM) exercise was allowed. The SCJ dislocation was treated conservatively with a figure-eight bandage for 6 weeks. Closed reduction was not attempted. At 12 months' follow-up, although the patient had mild discomfort around the ACJ while lifting a heavy object with the affected limb and slight anterior protrusion of the SCJ was still observed, he had regained full ROM and completely returned to his previous work. He was highly satisfied with the treatment.

### 2.2. Case 2

**Figure 3:**
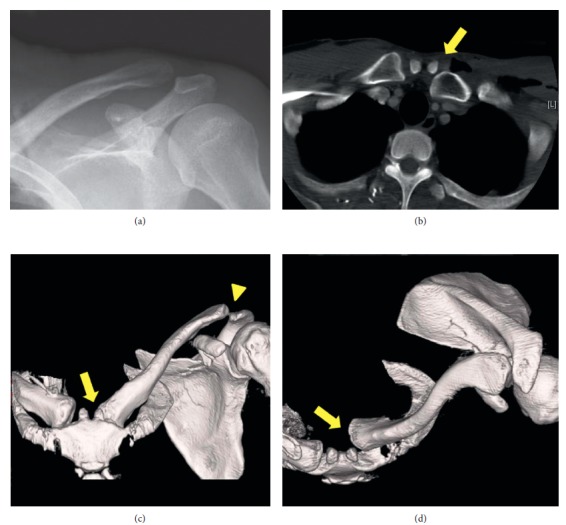
Case 2: images at the first presentation. (a) A radiograph shows type III ACJ dislocation. (b) A computed tomography (CT) scan shows posterior SCJ dislocation (arrow) as well as massive subcutaneous emphysema. (c) and (d) Three-dimensional reconstructed CT images of the left clavicle, which were made after the diagnosis was confirmed, clearly show ACJ dislocation (arrowhead) and posterior SCJ dislocation (arrow).

A 36-year-old East Asian, right hand-dominant male factory worker was transported to a trauma center after his upper body was accidentally compressed in a bag-making machine. He was diagnosed with a depressed skull fracture, acute epidural hematoma, left hemopneumothorax that required placement of a thoracostomy tube, massive subcutaneous emphysema, left coracoid process fracture, left scapula body fracture, and type III superior dislocation of the left ACJ. The initial CT scan also showed posterior dislocation of the ipsilateral SCJ (Figure 3), but an SCJ lesion was overlooked in the emergency department. He did not have neurovascular symptoms or airway compromise.

**Figure 4:**
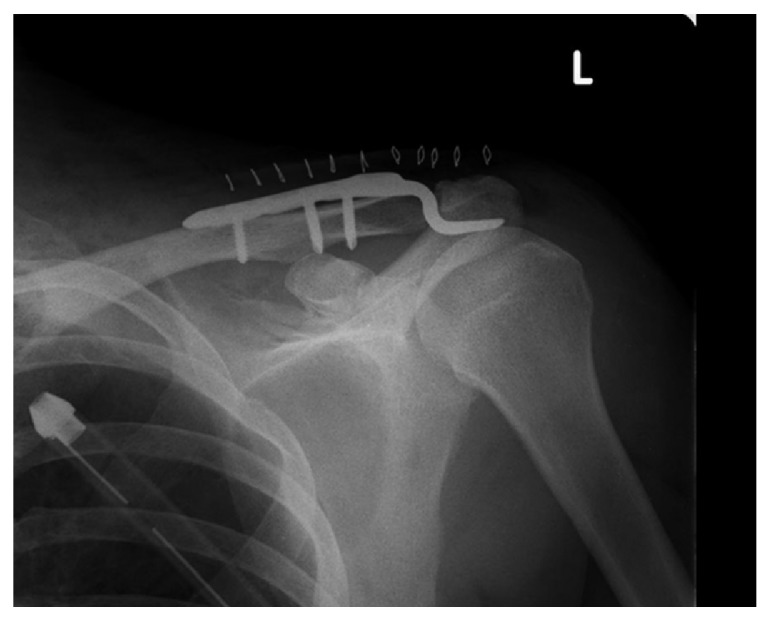
Case 2: a radiograph taken after ACJ surgery.

**Figure 5:**
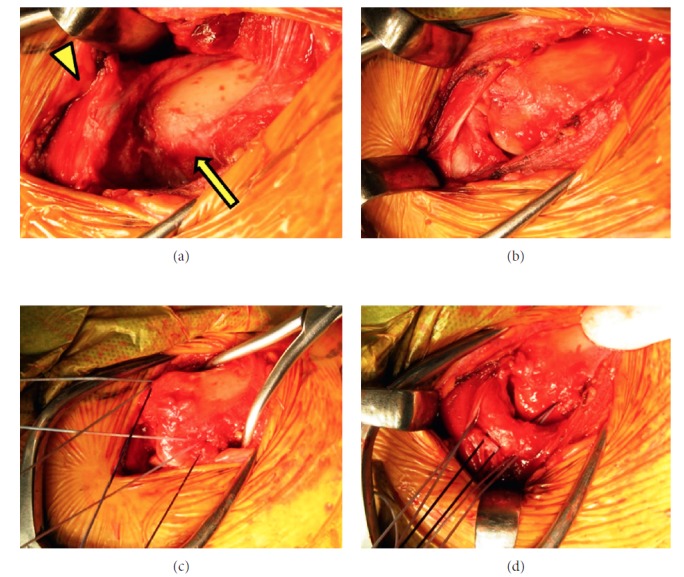
Case 2: intraoperative photographs of open reduction and augmentation of the SCJ. (a) Before reduction, the clavicle (arrow) was located behind the manubrium (arrowhead). (b) After reduction, the clavicle could maintain its position but was easily redislocated. (c) Three FiberWires were passed through a hole in the clavicle. (d) Sutures were also passed through holes in the manubrium and fastened to the surrounding soft tissue.

**Figure 6:**
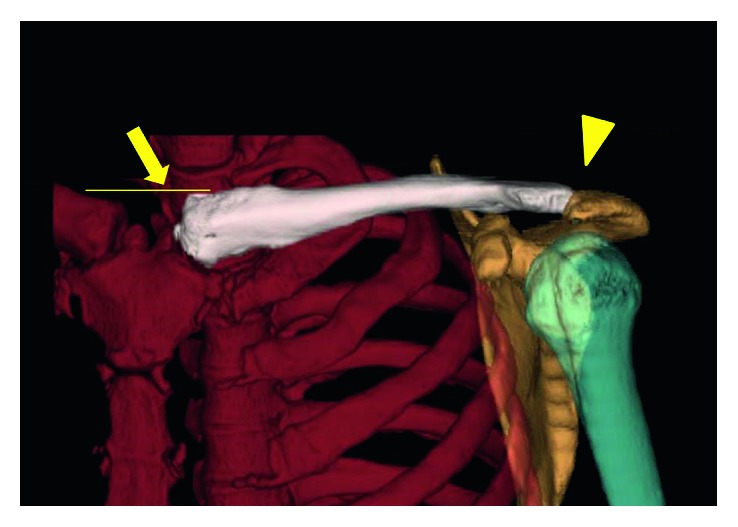
Case 2: a three-dimensional reconstructed CT scan of the SCJ at 12 months' follow-up shows a reduced ACJ (arrowhead) and SCJ (arrow) with remaining 2.5 mm superior displacement of the clavicle (line).

An emergency operation for the head injury was performed, and the ACJ injury was simultaneously fixed with a hook plate (Figure 4). On the second day after the procedure, SCJ dislocation was noticed during a radiological review. Closed reduction with a clamp was attempted, but grasping the clavicle was impossible due to excessive subcutaneous emphysema. The patient underwent open reduction. The reduction was performed without any difficulty by directly holding the clavicle with a clamp. The clavicle's position could be maintained without any support, but it was easily redislocated when compression force was applied to the medial clavicle. Surgical augmentation with reinforced, braided, polyethylene-blended sutures (FiberWire®, Arthrex, Naples, FL, USA) was performed. Three sutures were passed through a drilled hole on the clavicle; then, holes were made in the manubrium, as Thomas et al. [18] described. Sutures were also passed through remnants of the ligaments and joint capsule, and all were fastened together (Figure 5). A sling was used for 3 weeks; then, full ROM exercise was allowed. At 3 months' follow-up, bony union of the left coracoid process was observed and the hook plate was removed. At 12 months' follow up, although a CT scan showed 2.5 mm residual superior displacement (Figure 6), it was not obvious on a physical examination. The patient did not have symptoms and completely returned to his previous work with full ROM.

## 3. Discussion

In English literature that has been published since 1980, only 25 patients with true bipolar dislocation were reported (Tables 1 and 2). Among these reports, most patients had superior or posterior ACJ dislocation (type III or IV, as described by Rockwood and Young [24]) and anterior SCJ dislocation. Only a few patients with other combinations have been reported. Bipolar, posterior SCJ dislocation is extremely rare and only three cases, including ours, have been found.

The mechanism of injury is still debatable. This injury is frequently associated with high-energy trauma. Some authors suggested that this injury was not caused by a single-direction force but combinations of multiple-direction forces. In 1984, Maruyama et al. [25] proposed that the first rib plays an important role in the pathophysiology of bipolar dislocation as a pivot point. Based on their theory, we hypothesized that bipolar dislocation with anterior SCJ dislocation is caused by posteromedial-directed force from the anterolateral surface of the shoulder or through the outstretched hand. The force pushes the clavicle onto the first rib, and the proximal part of the clavicle is elevated due to this leverage motion, causing anterior SCJ dislocation. The same force or an additional force around the acromion, which can occur due to falling on the shoulder, also causes superior and/or posterior ACJ dislocation. The authors of that study also suggested that bipolar posterior SCJ dislocation was produced by a direct blow toward the proximal part of the clavicle from the anterior part; in addition, the clavicle is pushed onto the first rib. Then, the laterally directed component of the initial force or another inferior-directed force that pushes the acromion down causes ACJ dislocation [25]. Because the lever arm was shorter in patients with posterior SCJ dislocation than in those with anterior SCJ dislocation, the authors stated that a greater force was needed to cause bipolar dislocation in those with posterior SCJ dislocation than in those with anterior SCJ dislocation, and it was more likely to be accompanied by a proximal clavicle fracture at the intersection with the first rib. Some patients have a medial clavicle fracture at the intersection of the first rib and ipsilateral ACJ dislocation [5, 26–28]. Even though it cannot account for the mechanism of all bipolar dislocations, we think that the “first rib pivot theory” can be used to explain the characteristics of bipolar dislocation as well as the occurrence of another type of “floating clavicle” such as medial clavicle fracture with ACJ dislocation.

Many authors reported that SCJ dislocation is frequently missed at the first diagnostic imaging examination [29, 30]. Although some authors introduced specialized plain radiographic projections to diagnose SCJ pathology [31], these projections are often difficult to obtain in acute settings, especially in cases of high-energy trauma, which limits the use of plain radiographs in such situations [32]. Currently, CT is thought to be the most valuable tool for the early diagnosis of bipolar dislocation [11, 29]. In fact, reports of isolated SCJ dislocation, as well as bipolar dislocation, have been increasing since 1980, and this may be related to the increasing use of CT examinations for trauma patients. Scapinelli reported that three-dimensional reconstruction was useful to evaluate the direction of each dislocation and concluded that it was an essential tool for preoperative planning [11]. We utilized this modality not only to plan the operations but also to assess residual displacement postoperatively in Case 2.

Regarding the treatment, many authors have previously discussed both fracture and dislocation simultaneously, but we believe these should be discussed separately because they have different clinical courses and potential consequences. Reviewing existing reports, we found that surgically treated patients showed good results regardless of the timing of presentation, preinjury function, or type of ACJ dislocation, even though a publication bias might have existed. On the other hand, conservative treatment appeared to be generally acceptable, as shown in the reported cases; however, in several cases, conservative treatment led to an unacceptable result. Most of the delayed-presentation cases had received conservative treatment beforehand, but they remained symptomatic for months. Regarding ACJ lesions, Sanders et al. reported the largest case series of bipolar dislocation: two conservatively treated patients and four surgically treated patients who had residual symptoms after conservative treatment [19]. They did not clarify the types of ACJ dislocation in all cases but stated that type IV ACJ dislocation was the most common. Schemitsch et al. presented two patients with late-presentation bipolar dislocation, both of whom had type IV ACJ dislocation [22]. Therefore, patients with type IV bipolar ACJ dislocation may receive a benefit from surgical treatment of the ACJ in the acute stage, as recommended for isolated ACJ dislocation. All fixation techniques appeared to be equally effective to treat ACJ dislocation, and no conclusion can be made for the best fixation technique with the current evidence.

The treatment of SCJ dislocation is still controversial, even for those with solitary dislocation. In most cases, anterior dislocation is often treated conservatively, with or without closed reduction. The redislocation rate after closed reduction was reported to be substantially high [33], but residual symptoms were usually mild and well tolerated, even without a reduction procedure. In patients with bipolar, anterior SCJ dislocation, surgery led to a good result, but those who had conservative treatment also showed that it had few functional disadvantages for the SCJ. Only cosmetic issues remained, as seen in Case 1. We think that conservative treatment may be enough to treat most anterior SCJ lesions with bipolar dislocation in the acute setting, and operative treatment should be reserved for chronic, symptomatic cases or those who cannot accept the residual deformity of the SCJ.

Posterior SCJ dislocation requires prompt reduction to prevent neurovascular or airway compression [34]. Closed reduction should be attempted first, usually with a clamp. Tepolt et al. conducted a meta-analysis about posterior SCJ dislocation among adolescent patients and reported that the success rate of closed reduction was higher if the procedure was performed within 48 hours than if it was performed after 48 hours (55.8% and 30.8%, resp.) [35]. Thus, reduction should be performed as soon as possible. In Case 2, closed reduction was not successful because massive subcutaneous emphysema made it impossible for the surgeon to grasp the clavicle with a clamp percutaneously. We think that open reduction should be chosen first in such cases to prevent further soft tissue damage. In most cases, the joint will be stabilized after reduction. Tepolt also reported that the results of closed reduction only and operative treatment for isolated posterior SCJ dislocation were equally good; full function without recurrence was obtained in 92.31% and 95.83% of patients, respectively.

An issue that remains is whether or not the results of isolated SCJ dislocation can be applied to the treatment of those with bipolar lesions, which might be more unstable than monopolar dislocation. Two currently available reports on patients with bipolar, posterior SCJ dislocation showed good results due to surgical treatment: one of them was treated in an acute setting and the other was a delayed-presentation case. Both patients underwent open reduction and ligamentous augmentation. For bipolar, posterior SCJ dislocation, open augmentation can be considered if open reduction is needed after failed closed reduction in the acute- and delayed-presentation settings. It is still unclear whether additional open augmentation of the SCJ should be performed after successful closed reduction. We think that the additional advantage would be small; therefore, the decision regarding surgery should be made based on the patient's preference. Surgeons must choose less-invasive, low-risk surgical techniques in such situations.

Many authors implemented various procedures for SCJ augmentation to prevent further recurrence or instability. The techniques they used included fixation with metal devices such as a Kirschner wire [11, 20, 21], cerclage wire [13], compression screw [1], T-plate [12], and hook plate [22], as well as ligamentous reconstruction with polyester fiber tape [4], polyester surgical mesh [7], muscle strip [36], and tendon graft [23]. Most of these devices were sufficient to prevent recurrence, but the SCJ has an ROM of a maximum of 40°, and rigid joint bridging fixation may compromise shoulder movement. Furthermore, metal devices such as a Kirschner wire possess substantial risks. Lyons et al. reviewed 37 reported cases of devastating complications that were caused by migrated Kirschner wires used for shoulder operations. Twenty-one patients had SCJ dislocation. They reported that 8 out of 37 patients died due to major vascular injury and the other 6 patients sustained cardiac tamponade [37]. They concluded that pointed implants should never be used for SCJ fixation. In addition, implant migration was also reported, even among patients with a screw or plate [38]. We recommend that any metal hardware should be avoided when treating SCJ lesions whenever possible because of the SCJ's proximity to the vital organs and the serious consequences of using metal hardware. Flexible, ligamentous reinforcement with a tendon graft or artificial substitute can safely provide enough stability. Various techniques have been introduced; however, ruptured ligaments and capsules are expected to heal in acute cases if the joint is protected from excessive movement. We choose the FiberWire to stabilize the SCJ. Adamcik et al. mentioned that they implemented FiberWires in patients with anterior or posterior SCJ dislocation, which yielded good results [39]. Four of five patients had acute SCJ dislocation. Stabilization with FiberWires is a relatively simple technique and less invasive than ligament reconstruction with grafts. We believe that this is a good option to treat patients with acute SCJ dislocation because when it is used, surgeons should mind not only the stability of the joint but also the integrity of the joint envelope to minimize the rate of late complications.

## 4. Conclusion

We reported two patients with bipolar dislocation of the clavicle. One had anterior SCJ dislocation and the other had posterior SCJ dislocation. Both patients were successfully treated with surgery. According to our experience and literature review, we recommend surgical treatment for patients with type IV ACJ lesions, those with SCJ lesions with unreducible posterior dislocation, and those with chronic, symptomatic injuries. For other injury patterns, both conservative and surgical treatments appeared to be equally effective, but a further study is needed to reach an agreeable conclusion.

## Figures and Tables

**Table 1:** Summary of reported patients who underwent conservative treatment.

First author	Year	Sex	Age (y)	ACJ dislocation (direction/Rockwood's type)	SCJ dislocation	Described result
Gearen [8]	1982	M	27	Inferior/NA	Anterior	Functionally good; residual deformity at the SCJ
Jain [3]	1984	M	77	Superior/(III)	(Anterior)	Good
Cook [15]	1987	M	20	Superior/III	Anterior	Functionally good; residual deformity at the SCJ
Sanders [19]	1990	F	67	Posterior/IV	Anterior	Occasional tenderness and deformity at the lateral end
Sanders [19]	1990	F	21	Undescribed/NA^∗^	Anterior	Symptomatic
Eni-Olotu [16]	1997	M	63	Inferior/(IV)	Superior^#^	Residual pain at the lateral end requiring surgery
Pang^+^ [14]	2003	M	19	Superior/II	Anterior	Occasional discomfort and residual deformity at both ends

NA: not applicable; parentheses mean that the author(s) did not directly mention the result, but it could be interpreted using the description and/or figures in the articles; ^+^the authors reported two patients, one of whom was excluded because he/she had a fracture; ^∗^the authors stated that type IV was the most common type of dislocation among their six cases; ^#^the authors did not mention otherwise; it was impossible to interpret the direction of the dislocation with their images.

**Table 2:** Summary of reported patients who underwent surgical treatment (above the line: acute presentation, below the line: delayed presentation).

First author	Year	Sex	Age (y)	ACJ dislocation		SCJ dislocation		Timing of surgery	Described result
				Direction/type	Treatment	Direction	Treatment		
Echo [10]	1988	M	20	Inferior/(III)	Surgical (modified Phemister)	Anterior	Conservative	Undescribed	Functionally good; residual deformity at the SCJ
Arenas [20]	1993	M	26	Inferior/NA	Surgical (K-wire)	Anterior	Surgical (K-wire)	Undescribed	Good
Le Huec^+^ [21]	1998	M	58	Posterior/III	Surgical (K-wire)	Anterior	Surgical (K-wire)	6 weeks	Good
Scapinelli [11]	2004	F	18	Superior/III	Surgical (Weber technique)	Anterior	Surgical (K-wire)	19 days	Good
Yurdakul [1]	2012	M	21	Superior/III	Surgical (compression screw)	Anterior	Surgical (compression screw)	21 days	Functionally good
Choo [4]	2012	M	48	Superior/V	Surgical (hook plate)	(Anterior)	Surgical (polyester tape)	Undescribed	Good
Jiang [12]	2012	F	41	Posterosuperior/NA	Surgical (K-wire)	Anterior	Surgical (T-plate)	Undescribed	Good
Schuh [13]	2012	M	23	Posterosuperior/IV	Surgical (K- and cerclage wires)	Anterior	Surgical (cerclage wire)	3 weeks	Functionally good
Thyagarajan [7]	2015	M	51	Superior/III	Surgical (polyester mesh)	Posterior	Surgical (polyester mesh)	3 weeks	Good
Okano (presenting)	2017	M	45	Superior/III	Surgical (modified Cadenat)	Anterior	Conservative	10 days	Functionally good; residual deformity at the SCJ
Okano (presenting)	2017	M	36	Superior/III	Surgical (hook plate)	Posterior	Surgical (FiberWire)	0 days (ACJ)/1 day (SCJ)	Good

Sanders [19]	1990	M	26	Undescribed/NA^∗^	Surgical (ligament transfer)	Anterior	Conservative	18 months	Functionally good
Sanders [19]	1990	M	35	Undescribed/NA^∗^	Surgical (ligament transfer)	Anterior	Conservative	13 months	Functionally good
Sanders [19]	1990	M	20	Undescribed/NA^∗^	Surgical (ligament transfer)	Anterior	Conservative	3 months	Functionally good
Sanders [19]	1990	M	41	Posterior/IV	Surgical (ligament transfer)	Anterior	Conservative	12 months	Functionally good
Argintar [9]	2011	M	55	Superior/NA	Surgical (claviculectomy)	Anterior	Surgical (claviculectomy)	2 years	Relieve of previous symptoms
Schemitsch [22]	2011	F	49	Posterior/(IV)	Surgical (hook plate)	Anterior	Surgical (hook plate)	8 months	Good
Schemitsch [22]	2011	F	42	Posterior/(IV)	Surgical (hook plate)	Anterior	Surgical (hook plate)	6 months	Good
Yin [23]	2012	M	39	Posterosuperior/V	Surgical (tendon allograft)	Posterior	Surgical (tendon allograft)	10 weeks	Good

NA: not applicable; K-wire: Kirschner wire; parentheses in the “direction/type” column mean that the author(s) did not mention the result directly, but it could be interpreted using the description and/or figures in the articles; ^+^the authors reported two patients, one of whom was excluded because he/she had a fracture; ^∗^the authors stated that type IV was the most common type of dislocation among their six patients.

## References

[1] Yurdakul E., Salt Ö., Uzun E., Doğar F., Güney A., Durukan P. (2012). Traumatic floating clavicle. *The American Journal of Emergency Medicine*.

[2] Sopu A., Green C., Molony D. (2015). Traumatic floating clavicle: a case report. *Journal of Orthopaedic Case Reports*.

[3] Jain A. S. (1984). Traumatic floating clavicle: a case report. *Journal of Bone & Joint Surgery, British Volume*.

[4] Choo C. Y., Wong H. Y., Nordin A. (2012). Traumatic floating clavicle: a case report. *Malaysian Orthopaedic Journal*.

[5] Serra J. T., Tomas J., Batalla L. (2011). Traumatic floating clavicle: a case report. *Journal of Orthopaedic Trauma*.

[6] Gouse M., Jacob K. M., Poonnoose P. M. (2013). Traumatic floating clavicle: a case report and literature review. *Case Reports in Orthopedics*.

[7] Thyagarajan D., Webb M., Wallace A. (2015). A rare case of floating clavicle and a novel technique for stabilizing the sternoclavicular joint. *Shoulder & Elbow*.

[8] Gearen P. F., Petty W. (1982). Panclavicular dislocation. Report of a case. *The Journal of Bone & Joint Surgery*.

[9] Argintar E., Holzman M., Gunther S. (2011). Bipolar clavicular dislocation. *Orthopedics*.

[10] Echo B. S., Donati R. B., Powell C. E. (1988). Bipolar clavicular dislocation treated surgically: a case report. *The Journal of Bone & Joint Surgery*.

[11] Scapinelli R. (2004). Bipolar dislocation of the clavicle: 3D CT imaging and delayed surgical correction of a case. *Archives of Orthopaedic and Trauma Surgery*.

[12] Jiang W., Lei G.-H., Gao S.-G., Li Y.-S. (2012). Bipolar dislocation of the clavicle. *Indian Journal of Orthopaedics*.

[13] Schuh A., Thonse C. N., Schmickal T., Kleine L. (2012). Operative treatment of bipolar clavicular dislocation: a case report. *Journal of Orthopaedic Case Reports*.

[14] Pang K. P., Yung S. W., Lee T. S., Pang C. E. (2003). Bipolar clavicular injury. *Medical Journal of Malaysia*.

[15] Cook F., Horowitz M. (1987). Bipolar clavicular dislocation. Report of a case. *The Journal of Bone & Joint Surgery*.

[16] Eni-Olotu D. O., Hobbs N. J. (1997). Floating clavicle–simultaneous dislocation of both ends of the clavicle. *Injury*.

[17] Cerciello S., Edwards T. B., Morris B. J., Cerciello G., Walch G. (2014). The treatment of type III acromioclavicular dislocations with a modified Cadenat procedure: surgical technique and mid-term results. *Archives of Orthopaedic and Trauma Surgery*.

[18] Thomas D. P., Williams P. R., Hoddinott H.C. (2000). A ‘safe’ surgical technique for stabilisation of the sternoclavicular joint: a cadaveric and clinical study. *Annals of The Royal College of Surgeons of England*.

[19] Sanders J. O., Lyons F. A., Rockwood C. A. (1990). Management of dislocations of both ends of the clavicle. *The Journal of Bone & Joint Surgery*.

[20] Arenas A. J., Pampliega T., Iglesias J. (1993). Surgical management of bipolar clavicular dislocation. *Acta Orthopaedica Belgica*.

[21] Le Huec J. C., Mc Bride J. T., Liquois F., Lesprit E., Le Rebeller A. (1998). Bipolar lesion of the clavicle. *European Journal of Orthopaedic Surgery & Traumatology*.

[22] Schemitsch L. A., Schemitsch E. H., McKee M. D. (2011). Bipolar clavicle injury: posterior dislocation of the acromioclavicular joint with anterior dislocation of the sternoclavicular joint: a report of two cases. *Journal of Shoulder and Elbow Surgery*.

[23] Yin B., Byram I. R., Levine W. N. (2012). Posterior dislocation of both ends of the clavicle treated with allograft tendon reconstruction: a case report. *Journal of Shoulder and Elbow Surgery*.

[24] Rockwood C. A., Young D. C. (1990). Disorders of the acromioclavicular joint. *The Shoulder*.

[25] Maruyama K., Sugawara R., Sano S. (1984). Similar case of panclavicular dislocation. *Katakansetsu*.

[26] Poggetti A., Novi M., Rosati M., Battistini P., Parchi P., Lisanti M. (2016). Unusual medial-end clavicle fracture combined with double disruption of the superior shoulder suspensory complex (SSSC): a case report in triathlon athlete. *Journal of Orthopaedic Case Reports*.

[27] Akpinar S., Hersekli M. A., Demirörs H. (2002). Fracture of the medial third of the clavicle and dislocation of the acromioclavicular joint. *Artroplast Artroskopk Cerrah*.

[28] Marjoram T. P., Chakrabarti A. (2015). Segmental clavicle fracture and acromio-clavicular joint disruption: an unusual case report. *Shoulder & Elbow*.

[29] Thomas D. P., Davies A., Hoddinott H. C. (1999). Posterior sternoclavicular dislocations–a diagnosis easily missed. *Annals of The Royal College of Surgeons of England*.

[30] Jacob M., Snashall J., Dorfman A., Shesser R. (2013). X-ray-negative posterior sternoclavicular dislocation after minor trauma. *The American Journal of Emergency Medicine*.

[31] Cope R., Riddervold H. O., Shore J. L., Sistrom C. L. (1991). Dislocations of the sternoclavicular joint: anatomic basis, etiologies, and radiologic diagnosis. *Journal of Orthopaedic Trauma*.

[32] Ernberg L. A., Potter H. G. (2003). Radiographic evaluation of the acromioclavicular and sternoclavicular joints. *Clinics in Sports Medicine*.

[33] Eskola A. (1986). Sternoclavicular dislocation. A plea for open treatment. *Acta Orthopaedica Scandinavica*.

[34] Jougon J. B., Lepront D. J., Dromer C. E. (1996). Posterior dislocation of the sternoclavicular joint leading to mediastinal compression. *The Annals of Thoracic Surgery*.

[35] Tepolt F., Carry P. M., Heyn P. C., Miller N. H. (2014). Posterior sternoclavicular joint injuries in the adolescent population: a meta-analysis. *The American Journal of Sports Medicine*.

[36] Booth C. M., Roper B. A. (1979). Chronic dislocation of the sternoclavicular joint: an operative repair. *Clinical Orthopaedics and Related Research*.

[37] Lyons F. A., Rockwood C. A. (1990). Migration of pins used in operations on the shoulder. *The Journal of Bone & Joint Surgery*.

[38] Solooki S., Vosoughi A. R. (2017). Incredible position of broken sliding dynamic hip screw implant in the medial of thigh. *Acta Orthopaedica et Traumatologica Turcica*.

[39] Adamcik S., Ahler M., Gioutsos K., Schmid R. A., Kocher G. J. (2017). Repair of sternoclavicular joint dislocations with FiberWire®. *Archives of Orthopaedic and Trauma Surgery*.

